# Dimethyl 3,3′-(phenyl­methyl­ene)bis­(1*H*-indole-2-carboxyl­ate)

**DOI:** 10.1107/S1600536813024471

**Published:** 2013-09-07

**Authors:** Hong-Shun Sun, Yu-Long Li, Ning Xu, Hong Xu, Ji-Dong Zhang

**Affiliations:** a Chemical Engineering Department, Nanjing College of Chemical Technology, Geguan Road No. 265 Nanjing, Nanjing 210048, People’s Republic of China

## Abstract

In the title compound, C_27_H_22_N_2_O_4_, the two indole ring systems are approximately perpendicular to each other, with a dihedral angle of 84.5 (5)° between their planes; the benzene ring is twisted with respect to the two indole ring systems at angles of 78.5 (5) and 86.5 (3)°. In the crystal, mol­ecules are linked by N—H⋯O hydrogen bonds, weak C—H⋯O and C—H⋯N hydrogen bonds, and C—H⋯π inter­actions into a three-dimensional supra­molecular architecture.

## Related literature

For applications of indole derivatives, see: Poter *et al.* (1977 ▶); Sundberg (1996 ▶); Chang *et al.* (1999 ▶); Ge *et al.* (1999 ▶); Ni (2008 ▶); Sun *et al.* (2012 ▶).
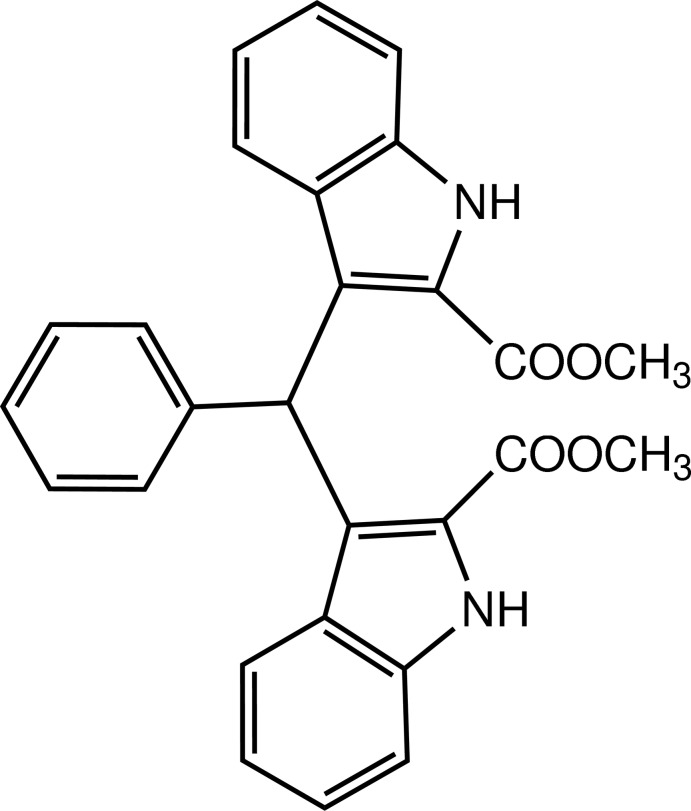


## Experimental

### 

#### Crystal data

C_27_H_22_N_2_O_4_
*M* *_r_* = 438.47
Monoclinic, 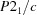

*a* = 13.604 (3) Å
*b* = 15.560 (3) Å
*c* = 11.274 (2) Å
β = 112.66 (3)°
*V* = 2202.2 (8) Å^3^
*Z* = 4
Mo *K*α radiation
μ = 0.09 mm^−1^
*T* = 293 K
0.30 × 0.20 × 0.10 mm

#### Data collection

Enraf–Nonis CAD-4 diffractometer
4196 measured reflections
4021 independent reflections
2322 reflections with *I* > 2σ(*I*)
*R*_int_ = 0.028
3 standard reflections every 200 reflections intensity decay: 1%

#### Refinement

*R*[*F* ^2^ > 2σ(*F* ^2^)] = 0.062
*wR*(*F* ^2^) = 0.181
*S* = 1.00
4021 reflections
298 parameters
H-atom parameters constrained
Δρ_max_ = 0.26 e Å^−3^
Δρ_min_ = −0.22 e Å^−3^

### 

Data collection: *CAD-4 EXPRESS* (Enraf–Nonius, 1994 ▶); cell refinement: *CAD-4 EXPRESS*; data reduction: *XCAD4* (Harms & Wocadlo, 1995 ▶); program(s) used to solve structure: *SHELXTL* (Sheldrick, 2008 ▶); program(s) used to refine structure: *SHELXTL*; molecular graphics: *SHELXTL*; software used to prepare material for publication: *SHELXTL*.

## Supplementary Material

Crystal structure: contains datablock(s) I, New_Global_Publ_Block. DOI: 10.1107/S1600536813024471/xu5723sup1.cif

Structure factors: contains datablock(s) I. DOI: 10.1107/S1600536813024471/xu5723Isup2.hkl

Supplementary material file. DOI: 10.1107/S1600536813024471/xu5723Isup3.cml

Additional supplementary materials:  crystallographic information; 3D view; checkCIF report

## Figures and Tables

**Table 1:** Hydrogen-bond geometry (Å, °) *Cg*1 and *Cg*4 are the centroids of the N1-pyrrole and C15-benzene rings, respectively.

*D*—H⋯*A*	*D*—H	H⋯*A*	*D*⋯*A*	*D*—H⋯*A*
N2—H2*A*⋯O2^i^	0.86	2.02	2.870 (4)	169
C11—H11*A*⋯O3^ii^	0.96	2.60	3.221 (4)	123
C11—H11*B*⋯N1^iii^	0.96	2.61	3.443 (5)	145
C11—H11*C*⋯O4^i^	0.96	2.53	3.333 (5)	142
C5—H5*A*⋯*Cg*4^iv^	0.93	2.76	3.659 (4)	164
C11—H11*B*⋯*Cg*1^iii^	0.96	2.55	3.366 (4)	143
C21—H21*B*⋯*Cg*4^i^	0.96	2.73	3.516 (5)	139

Symmetry codes: (i) 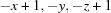; (ii) 
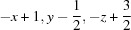
; (iii) 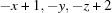; (iv) 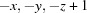.

**Fig. 1.**
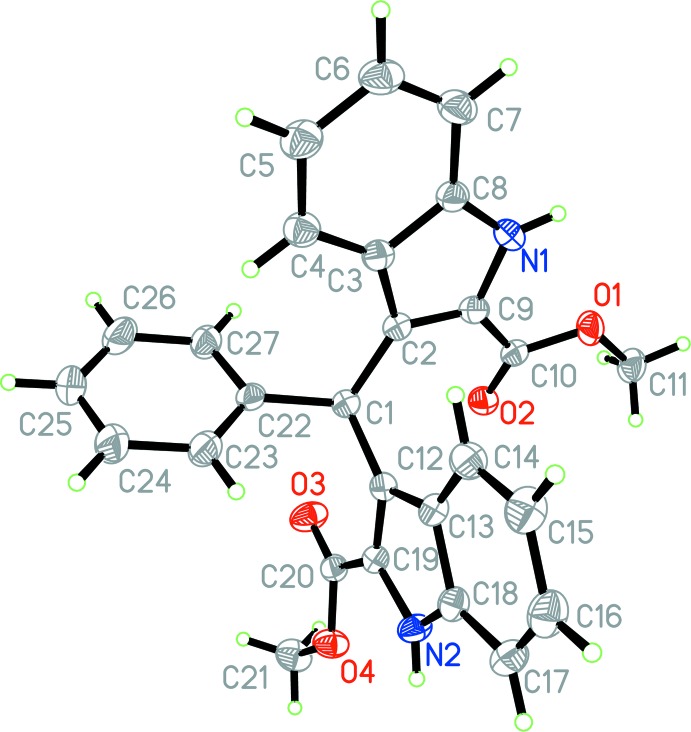
The molecular structure of (I), showing the atom-numbering scheme and displacement ellipsoids at the 30% probability level.

**Fig. 2.**
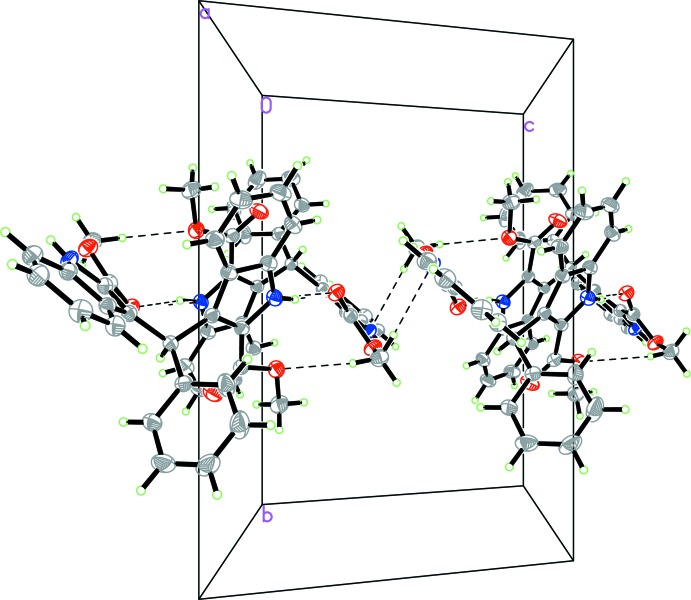
A packing diagram of (I). Intermolecular hydrogen bonds are shown as dashed lines.

## supplementary crystallographic information

### 1. Comment

Indole derivatives are found abundantly in a variety of natural plants and exhibit various physiological properties (Poter *et al.*, 1977; Sundberg, 1996). Among them, bis-indolymethane derivatives are found to be kinds of potentially bioactive compounds (Chang *et al.*, 1999; Ge *et al.*, 1999). In recent years, the synthesis and application of bis-indolymethane derivatives have been widely studied. The title compound is one of bis-indolymethane derivatives as a precursor for MRI Contrast Agents(Ni, 2008). We report here its crystal structure.

The molecular structure of the title compound is shown in Fig. 1. The benzene ring is twisted to the two indole rings with the dihedral angles of 101.5 (5) and 93.5 (3)°, respectively. Two indole rings make a dihedral angle of 84.5 (5)° to each other.

As shown in Figure 2, the molecules are linked by N—H···O and C—H···O and C—H···N hydrogen bonds into dimers in the crystal lattice. The structural parameters for the intermolecular hydrogen bonds resulting in the formation of dimers are given in Table 1.

### 2. Experimental

Methyl indole-2-carboxylate (17.5 g, 100 mmol) was dissolved in 200 ml methanol; commercially available benzaldehyde (5.3 g, 50 mmol) was added and the mixture was heated to reflux temperature. Concentrated HCl (3.7 ml) was added and the reaction was left for 1 h. After cooling the white product was filtered off and washed thoroughly with methanol. The reaction can be followed by TLC (CHCl_3_:hexane = 1:1). Yield was 93%. Crystals of the title compound suitable for X-ray analysis were obtained by slow evaporation of an ethanol solution.

### 3. Refinement

H atoms were positioned geometrically, with N— H = 0.86 Å and C—H = 0.93, 0.96, and 0.98 Å for aromatic, methyl, and methine H, respectively, and constrained to ride on their parent atoms, with *U*_iso_(H) = xU_eq_(C,N), where *x* = 1.5 for methyl H, and *x* = 1.2 for all other H atoms.

### Crystal data

**Table:**

C_27_H_22_N_2_O_4_	*F*(000) = 920
*M**_r_* = 438.47	*D*_x_ = 1.322 Mg m^−^^3^
Monoclinic, *P*2_1_/*c*	Mo *K*α radiation, λ = 0.71073 Å
Hall symbol: -P 2ybc	Cell parameters from 25 reflections
*a* = 13.604 (3) Å	θ = 9–13°
*b* = 15.560 (3) Å	µ = 0.09 mm^−^^1^
*c* = 11.274 (2) Å	*T* = 293 K
β = 112.66 (3)°	Block, colorless
*V* = 2202.2 (8) Å^3^	0.30 × 0.20 × 0.10 mm
*Z* = 4	

### Data collection

**Table:**

Enraf–Nonis CAD-4 diffractometer	*R*_int_ = 0.028
Radiation source: fine-focus sealed tube	θ_max_ = 25.4°, θ_min_ = 1.6°
Graphite monochromator	*h* = −16→0
ω/2θ scans	*k* = 0→18
4196 measured reflections	*l* = −12→13
4021 independent reflections	3 standard reflections every 200 reflections
2322 reflections with *I* > 2σ(*I*)	intensity decay: 1%

### Refinement

**Table:**

Refinement on *F*^2^	Primary atom site location: structure-invariant direct methods
Least-squares matrix: full	Secondary atom site location: difference Fourier map
*R*[*F*^2^ > 2σ(*F*^2^)] = 0.062	Hydrogen site location: inferred from neighbouring sites
*wR*(*F*^2^) = 0.181	H-atom parameters constrained
*S* = 1.00	*w* = 1/[σ^2^(*F*_o_^2^) + (0.088*P*)^2^] where *P* = (*F*_o_^2^ + 2*F*_c_^2^)/3
4021 reflections	(Δ/σ)_max_ < 0.001
298 parameters	Δρ_max_ = 0.26 e Å^−^^3^
0 restraints	Δρ_min_ = −0.22 e Å^−^^3^

### Special details

**Table:**

Geometry. All e.s.d.'s (except the e.s.d. in the dihedral angle between two l.s. planes) are estimated using the full covariance matrix. The cell e.s.d.'s are taken into account individually in the estimation of e.s.d.'s in distances, angles and torsion angles; correlations between e.s.d.'s in cell parameters are only used when they are defined by crystal symmetry. An approximate (isotropic) treatment of cell e.s.d.'s is used for estimating e.s.d.'s involving l.s. planes.
Refinement. Refinement of *F*^2^ against ALL reflections. The weighted *R*-factor *wR* and goodness of fit *S* are based on *F*^2^, conventional *R*-factors *R* are based on *F*, with *F* set to zero for negative *F*^2^. The threshold expression of *F*^2^ > σ(*F*^2^) is used only for calculating *R*-factors(gt) *etc*. and is not relevant to the choice of reflections for refinement. *R*-factors based on *F*^2^ are statistically about twice as large as those based on *F*, and *R*- factors based on ALL data will be even larger.

### Fractional atomic coordinates and isotropic or equivalent isotropic displacement parameters (Å^2^)

**Table:**

	*x*	*y*	*z*	*U*_iso_*/*U*_eq_	
O1	0.52156 (18)	−0.10234 (15)	0.9162 (2)	0.0504 (7)	
N1	0.3221 (2)	−0.07271 (18)	0.8973 (3)	0.0470 (8)	
H1A	0.3563	−0.1099	0.9548	0.056*	
C1	0.3029 (2)	0.08122 (19)	0.6386 (3)	0.0351 (7)	
H1B	0.3709	0.1108	0.6826	0.042*	
O2	0.52350 (18)	0.01567 (15)	0.8016 (2)	0.0478 (6)	
N2	0.3660 (2)	−0.00463 (17)	0.3694 (3)	0.0422 (7)	
H2A	0.3961	−0.0012	0.3150	0.051*	
C2	0.2866 (3)	0.0248 (2)	0.7391 (3)	0.0371 (8)	
O3	0.4526 (2)	0.18689 (16)	0.5589 (3)	0.0593 (7)	
C3	0.1909 (3)	0.0105 (2)	0.7638 (3)	0.0377 (8)	
O4	0.46982 (19)	0.14311 (15)	0.3787 (2)	0.0502 (6)	
C4	0.0870 (3)	0.0436 (2)	0.7147 (3)	0.0456 (9)	
H4A	0.0657	0.0822	0.6464	0.055*	
C5	0.0171 (3)	0.0181 (2)	0.7694 (4)	0.0539 (10)	
H5A	−0.0517	0.0403	0.7376	0.065*	
C6	0.0467 (3)	−0.0403 (2)	0.8713 (4)	0.0545 (10)	
H6A	−0.0024	−0.0558	0.9064	0.065*	
C7	0.1462 (3)	−0.0749 (2)	0.9201 (4)	0.0533 (10)	
H7A	0.1659	−0.1139	0.9878	0.064*	
C8	0.2178 (3)	−0.0499 (2)	0.8649 (3)	0.0411 (8)	
C9	0.3638 (3)	−0.0263 (2)	0.8228 (3)	0.0397 (8)	
C10	0.4765 (3)	−0.0341 (2)	0.8434 (3)	0.0417 (8)	
C11	0.6316 (3)	−0.1198 (2)	0.9398 (4)	0.0545 (10)	
H11A	0.6544	−0.1702	0.9924	0.082*	
H11B	0.6746	−0.0717	0.9832	0.082*	
H11C	0.6390	−0.1290	0.8595	0.082*	
C12	0.3159 (2)	0.02960 (19)	0.5322 (3)	0.0346 (7)	
C13	0.2685 (2)	−0.0511 (2)	0.4786 (3)	0.0373 (8)	
C14	0.1994 (3)	−0.1099 (2)	0.5024 (3)	0.0462 (9)	
H14A	0.1743	−0.0993	0.5669	0.055*	
C15	0.1699 (3)	−0.1824 (2)	0.4296 (4)	0.0556 (10)	
H15A	0.1243	−0.2211	0.4454	0.067*	
C16	0.2062 (3)	−0.2005 (2)	0.3313 (4)	0.0570 (10)	
H16A	0.1843	−0.2506	0.2833	0.068*	
C17	0.2733 (3)	−0.1452 (2)	0.3054 (3)	0.0485 (9)	
H17A	0.2985	−0.1573	0.2413	0.058*	
C18	0.3028 (2)	−0.0704 (2)	0.3775 (3)	0.0402 (8)	
C19	0.3741 (2)	0.0555 (2)	0.4624 (3)	0.0360 (7)	
C20	0.4351 (3)	0.1350 (2)	0.4750 (3)	0.0394 (8)	
C21	0.5280 (3)	0.2203 (3)	0.3776 (4)	0.0608 (11)	
H21A	0.5483	0.2193	0.3050	0.091*	
H21B	0.5907	0.2235	0.4553	0.091*	
H21C	0.4838	0.2695	0.3718	0.091*	
C22	0.2189 (3)	0.1521 (2)	0.5862 (3)	0.0380 (8)	
C23	0.1452 (3)	0.1525 (3)	0.4629 (4)	0.0649 (12)	
H23A	0.1441	0.1072	0.4087	0.078*	
C24	0.0724 (4)	0.2182 (3)	0.4161 (4)	0.0899 (17)	
H24A	0.0223	0.2164	0.3321	0.108*	
C25	0.0744 (4)	0.2856 (3)	0.4939 (5)	0.0797 (14)	
H25A	0.0268	0.3309	0.4626	0.096*	
C26	0.1465 (4)	0.2865 (3)	0.6181 (5)	0.0688 (12)	
H26A	0.1469	0.3319	0.6719	0.083*	
C27	0.2187 (3)	0.2202 (2)	0.6638 (4)	0.0493 (9)	
H27A	0.2679	0.2216	0.7484	0.059*	

### Atomic displacement parameters (Å^2^)

**Table:**

	*U*^11^	*U*^22^	*U*^33^	*U*^12^	*U*^13^	*U*^23^
O1	0.0438 (14)	0.0483 (15)	0.0580 (15)	0.0155 (12)	0.0183 (12)	0.0135 (12)
N1	0.0547 (19)	0.0451 (18)	0.0455 (17)	0.0053 (14)	0.0240 (15)	0.0124 (14)
C1	0.0370 (17)	0.0347 (18)	0.0341 (17)	0.0010 (14)	0.0144 (14)	−0.0007 (14)
O2	0.0478 (14)	0.0516 (15)	0.0512 (14)	0.0058 (12)	0.0270 (12)	0.0081 (12)
N2	0.0465 (16)	0.0447 (17)	0.0440 (16)	−0.0025 (14)	0.0270 (14)	−0.0041 (14)
C2	0.0411 (19)	0.0375 (18)	0.0357 (17)	0.0040 (15)	0.0180 (15)	−0.0005 (15)
O3	0.082 (2)	0.0447 (15)	0.0680 (17)	−0.0167 (14)	0.0468 (16)	−0.0162 (14)
C3	0.0401 (19)	0.0353 (18)	0.0395 (18)	−0.0035 (15)	0.0174 (15)	−0.0030 (15)
O4	0.0603 (16)	0.0501 (15)	0.0481 (14)	−0.0144 (12)	0.0296 (12)	−0.0017 (12)
C4	0.042 (2)	0.049 (2)	0.046 (2)	−0.0019 (17)	0.0178 (17)	0.0003 (17)
C5	0.042 (2)	0.059 (2)	0.065 (2)	−0.0045 (18)	0.0251 (19)	−0.001 (2)
C6	0.057 (2)	0.054 (2)	0.063 (2)	−0.014 (2)	0.036 (2)	−0.006 (2)
C7	0.066 (3)	0.045 (2)	0.058 (2)	−0.007 (2)	0.034 (2)	0.0022 (19)
C8	0.045 (2)	0.0383 (19)	0.0454 (19)	−0.0016 (16)	0.0233 (16)	−0.0006 (16)
C9	0.046 (2)	0.0394 (19)	0.0384 (18)	0.0004 (16)	0.0211 (16)	0.0003 (15)
C10	0.051 (2)	0.041 (2)	0.0364 (18)	0.0078 (17)	0.0204 (17)	−0.0025 (16)
C11	0.047 (2)	0.054 (2)	0.058 (2)	0.0163 (18)	0.0154 (19)	0.0062 (19)
C12	0.0355 (17)	0.0329 (17)	0.0352 (17)	0.0026 (14)	0.0135 (14)	0.0023 (14)
C13	0.0368 (18)	0.0350 (18)	0.0411 (18)	0.0036 (15)	0.0161 (15)	−0.0015 (15)
C14	0.048 (2)	0.043 (2)	0.051 (2)	−0.0051 (17)	0.0229 (18)	0.0006 (17)
C15	0.050 (2)	0.047 (2)	0.072 (3)	−0.0076 (18)	0.025 (2)	−0.005 (2)
C16	0.057 (2)	0.043 (2)	0.064 (3)	−0.0056 (19)	0.015 (2)	−0.0113 (19)
C17	0.051 (2)	0.048 (2)	0.047 (2)	0.0008 (18)	0.0193 (18)	−0.0101 (18)
C18	0.0360 (18)	0.0386 (19)	0.0448 (19)	0.0019 (15)	0.0140 (15)	−0.0013 (16)
C19	0.0399 (18)	0.0335 (18)	0.0370 (17)	0.0037 (15)	0.0174 (15)	0.0006 (15)
C20	0.0399 (19)	0.039 (2)	0.043 (2)	0.0038 (15)	0.0203 (16)	0.0043 (17)
C21	0.067 (3)	0.057 (2)	0.064 (3)	−0.016 (2)	0.032 (2)	0.005 (2)
C22	0.0425 (19)	0.0363 (18)	0.0433 (19)	0.0023 (15)	0.0256 (16)	0.0028 (15)
C23	0.070 (3)	0.078 (3)	0.042 (2)	0.033 (2)	0.016 (2)	−0.003 (2)
C24	0.100 (4)	0.116 (4)	0.047 (3)	0.064 (3)	0.021 (2)	0.011 (3)
C25	0.100 (4)	0.073 (3)	0.079 (3)	0.048 (3)	0.049 (3)	0.028 (3)
C26	0.087 (3)	0.049 (3)	0.084 (3)	0.018 (2)	0.049 (3)	−0.002 (2)
C27	0.054 (2)	0.043 (2)	0.051 (2)	0.0056 (18)	0.0204 (18)	−0.0040 (18)

### Geometric parameters (Å, º)

**Table:**

O1—C10	1.338 (4)	C11—H11B	0.9600
O1—C11	1.442 (4)	C11—H11C	0.9600
N1—C8	1.369 (4)	C12—C19	1.375 (4)
N1—C9	1.385 (4)	C12—C13	1.435 (4)
N1—H1A	0.8600	C13—C14	1.410 (4)
C1—C12	1.510 (4)	C13—C18	1.420 (4)
C1—C2	1.516 (4)	C14—C15	1.361 (5)
C1—C22	1.532 (4)	C14—H14A	0.9300
C1—H1B	0.9800	C15—C16	1.405 (5)
O2—C10	1.210 (4)	C15—H15A	0.9300
N2—C18	1.363 (4)	C16—C17	1.366 (5)
N2—C19	1.378 (4)	C16—H16A	0.9300
N2—H2A	0.8600	C17—C18	1.387 (5)
C2—C9	1.364 (4)	C17—H17A	0.9300
C2—C3	1.450 (4)	C19—C20	1.465 (5)
O3—C20	1.196 (4)	C21—H21A	0.9600
C3—C4	1.402 (4)	C21—H21B	0.9600
C3—C8	1.412 (4)	C21—H21C	0.9600
O4—C20	1.346 (4)	C22—C23	1.365 (5)
O4—C21	1.441 (4)	C22—C27	1.376 (5)
C4—C5	1.376 (5)	C23—C24	1.378 (5)
C4—H4A	0.9300	C23—H23A	0.9300
C5—C6	1.398 (5)	C24—C25	1.361 (6)
C5—H5A	0.9300	C24—H24A	0.9300
C6—C7	1.360 (5)	C25—C26	1.365 (6)
C6—H6A	0.9300	C25—H25A	0.9300
C7—C8	1.398 (5)	C26—C27	1.381 (5)
C7—H7A	0.9300	C26—H26A	0.9300
C9—C10	1.465 (5)	C27—H27A	0.9300
C11—H11A	0.9600		
			
C10—O1—C11	117.4 (3)	C13—C12—C1	129.0 (3)
C8—N1—C9	109.1 (3)	C14—C13—C18	117.7 (3)
C8—N1—H1A	125.5	C14—C13—C12	135.4 (3)
C9—N1—H1A	125.5	C18—C13—C12	106.9 (3)
C12—C1—C2	112.4 (2)	C15—C14—C13	119.1 (3)
C12—C1—C22	111.9 (3)	C15—C14—H14A	120.4
C2—C1—C22	113.8 (3)	C13—C14—H14A	120.4
C12—C1—H1B	106.0	C14—C15—C16	122.0 (3)
C2—C1—H1B	106.0	C14—C15—H15A	119.0
C22—C1—H1B	106.0	C16—C15—H15A	119.0
C18—N2—C19	109.1 (3)	C17—C16—C15	120.7 (3)
C18—N2—H2A	125.4	C17—C16—H16A	119.7
C19—N2—H2A	125.4	C15—C16—H16A	119.7
C9—C2—C3	106.7 (3)	C16—C17—C18	118.0 (3)
C9—C2—C1	124.0 (3)	C16—C17—H17A	121.0
C3—C2—C1	129.3 (3)	C18—C17—H17A	121.0
C4—C3—C8	118.1 (3)	N2—C18—C17	129.7 (3)
C4—C3—C2	135.4 (3)	N2—C18—C13	107.8 (3)
C8—C3—C2	106.5 (3)	C17—C18—C13	122.5 (3)
C20—O4—C21	116.7 (3)	C12—C19—N2	110.0 (3)
C5—C4—C3	118.9 (3)	C12—C19—C20	128.5 (3)
C5—C4—H4A	120.6	N2—C19—C20	121.5 (3)
C3—C4—H4A	120.6	O3—C20—O4	123.3 (3)
C4—C5—C6	121.8 (4)	O3—C20—C19	125.4 (3)
C4—C5—H5A	119.1	O4—C20—C19	111.3 (3)
C6—C5—H5A	119.1	O4—C21—H21A	109.5
C7—C6—C5	121.1 (3)	O4—C21—H21B	109.5
C7—C6—H6A	119.5	H21A—C21—H21B	109.5
C5—C6—H6A	119.5	O4—C21—H21C	109.5
C6—C7—C8	117.8 (3)	H21A—C21—H21C	109.5
C6—C7—H7A	121.1	H21B—C21—H21C	109.5
C8—C7—H7A	121.1	C23—C22—C27	117.6 (3)
N1—C8—C7	129.6 (3)	C23—C22—C1	122.9 (3)
N1—C8—C3	107.9 (3)	C27—C22—C1	119.5 (3)
C7—C8—C3	122.4 (3)	C22—C23—C24	122.1 (4)
C2—C9—N1	109.7 (3)	C22—C23—H23A	118.9
C2—C9—C10	129.7 (3)	C24—C23—H23A	118.9
N1—C9—C10	120.6 (3)	C25—C24—C23	119.5 (4)
O2—C10—O1	124.1 (3)	C25—C24—H24A	120.3
O2—C10—C9	124.3 (3)	C23—C24—H24A	120.3
O1—C10—C9	111.6 (3)	C24—C25—C26	119.8 (4)
O1—C11—H11A	109.5	C24—C25—H25A	120.1
O1—C11—H11B	109.5	C26—C25—H25A	120.1
H11A—C11—H11B	109.5	C25—C26—C27	120.1 (4)
O1—C11—H11C	109.5	C25—C26—H26A	119.9
H11A—C11—H11C	109.5	C27—C26—H26A	119.9
H11B—C11—H11C	109.5	C22—C27—C26	120.9 (4)
C19—C12—C13	106.3 (3)	C22—C27—H27A	119.5
C19—C12—C1	124.6 (3)	C26—C27—H27A	119.5
			
C12—C1—C2—C9	69.5 (4)	C19—C12—C13—C18	0.7 (3)
C22—C1—C2—C9	−161.9 (3)	C1—C12—C13—C18	177.6 (3)
C12—C1—C2—C3	−107.8 (4)	C18—C13—C14—C15	0.9 (5)
C22—C1—C2—C3	20.8 (5)	C12—C13—C14—C15	179.8 (3)
C9—C2—C3—C4	178.2 (4)	C13—C14—C15—C16	0.0 (5)
C1—C2—C3—C4	−4.2 (6)	C14—C15—C16—C17	0.1 (6)
C9—C2—C3—C8	0.4 (4)	C15—C16—C17—C18	−1.0 (5)
C1—C2—C3—C8	178.1 (3)	C19—N2—C18—C17	−179.1 (3)
C8—C3—C4—C5	2.0 (5)	C19—N2—C18—C13	0.5 (4)
C2—C3—C4—C5	−175.6 (3)	C16—C17—C18—N2	−178.5 (3)
C3—C4—C5—C6	−0.6 (5)	C16—C17—C18—C13	2.0 (5)
C4—C5—C6—C7	−0.5 (6)	C14—C13—C18—N2	178.5 (3)
C5—C6—C7—C8	0.2 (6)	C12—C13—C18—N2	−0.7 (3)
C9—N1—C8—C7	−175.0 (3)	C14—C13—C18—C17	−1.9 (5)
C9—N1—C8—C3	2.1 (4)	C12—C13—C18—C17	178.9 (3)
C6—C7—C8—N1	178.0 (3)	C13—C12—C19—N2	−0.4 (4)
C6—C7—C8—C3	1.3 (5)	C1—C12—C19—N2	−177.5 (3)
C4—C3—C8—N1	−179.8 (3)	C13—C12—C19—C20	177.8 (3)
C2—C3—C8—N1	−1.5 (4)	C1—C12—C19—C20	0.6 (5)
C4—C3—C8—C7	−2.4 (5)	C18—N2—C19—C12	−0.1 (4)
C2—C3—C8—C7	175.8 (3)	C18—N2—C19—C20	−178.4 (3)
C3—C2—C9—N1	0.8 (4)	C21—O4—C20—O3	−1.9 (5)
C1—C2—C9—N1	−177.0 (3)	C21—O4—C20—C19	178.0 (3)
C3—C2—C9—C10	−176.8 (3)	C12—C19—C20—O3	8.2 (6)
C1—C2—C9—C10	5.4 (5)	N2—C19—C20—O3	−173.9 (3)
C8—N1—C9—C2	−1.8 (4)	C12—C19—C20—O4	−171.7 (3)
C8—N1—C9—C10	176.0 (3)	N2—C19—C20—O4	6.3 (4)
C11—O1—C10—O2	−3.5 (5)	C12—C1—C22—C23	17.2 (5)
C11—O1—C10—C9	177.5 (3)	C2—C1—C22—C23	−111.7 (4)
C2—C9—C10—O2	12.8 (6)	C12—C1—C22—C27	−160.6 (3)
N1—C9—C10—O2	−164.6 (3)	C2—C1—C22—C27	70.6 (4)
C2—C9—C10—O1	−168.2 (3)	C27—C22—C23—C24	−0.1 (6)
N1—C9—C10—O1	14.4 (4)	C1—C22—C23—C24	−177.9 (4)
C2—C1—C12—C19	−150.7 (3)	C22—C23—C24—C25	1.0 (8)
C22—C1—C12—C19	79.7 (4)	C23—C24—C25—C26	−1.7 (8)
C2—C1—C12—C13	32.8 (4)	C24—C25—C26—C27	1.4 (7)
C22—C1—C12—C13	−96.8 (4)	C23—C22—C27—C26	−0.1 (5)
C19—C12—C13—C14	−178.3 (4)	C1—C22—C27—C26	177.8 (3)
C1—C12—C13—C14	−1.3 (6)	C25—C26—C27—C22	−0.5 (6)

### Hydrogen-bond geometry (Å, º)

Cg1 and cg4 are the centroids of the N1-pyrrole and C15-benzene rings, respectively.

**Table:**

*D*—H···*A*	*D*—H	H···*A*	*D*···*A*	*D*—H···*A*
N2—H2*A*···O2^i^	0.86	2.02	2.870 (4)	169
C11—H11*A*···O3^ii^	0.96	2.60	3.221 (4)	123
C11—H11*B*···N1^iii^	0.96	2.61	3.443 (5)	145
C11—H11*C*···O4^i^	0.96	2.53	3.333 (5)	142
C5—H5*A*···*Cg*4^iv^	0.93	2.76	3.659 (4)	164
C11—H11*B*···*Cg*1^iii^	0.96	2.55	3.366 (4)	143
C21—H21*B*···*Cg*4^i^	0.96	2.73	3.516 (5)	139

Symmetry codes: (i) −*x*+1, −*y*, −*z*+1; (ii) −*x*+1, *y*−1/2, −*z*+3/2; (iii) −*x*+1, −*y*, −*z*+2; (iv) −*x*, −*y*, −*z*+1.
